# Evaluating the oncological safety of neoadjuvant chemotherapy in locally advanced colon carcinoma: a systematic review and meta-analysis of randomised clinical trials and propensity-matched studies

**DOI:** 10.1007/s00384-023-04482-x

**Published:** 2023-07-11

**Authors:** Matthew G. Davey, Amira H. Amir, Odhrán K. Ryan, Mark Donnelly, Noel E. Donlon, Mark Regan, Babak Meshkat, Emmeline Nugent, Myles Joyce, Aisling M. Hogan

**Affiliations:** 1 Royal College of Surgeons in Ireland, 123 St Stephen’s Green, Dublin 2, Dublin, Ireland; 2 Department of Surgery, Galway University Hospitals, Galway, H91 YRY71 Ireland

**Keywords:** Colon cancer, Neoadjuvant therapies, Personalised medicine

## Abstract

**Purpose:**

Use of neoadjuvant chemotherapy (NAC) for locally advanced colon cancer (LACC) remains controversial. An integrated analysis of data from high-quality studies may inform the long-term safety of NAC for this cohort. Our aim was to perform a systematic review and meta-analysis of randomised clinical trials (RCTs) and propensity-matched studies to assess the oncological safety of NAC in patients with LACC.

**Methods:**

A systematic review was performed as per preferred reporting items for systematic reviews and meta-analyses (PRISMA) guidelines. Survival was expressed as hazard ratios using time-to-effect generic inverse variance methodology, while surgical outcomes were expressed as odds ratios (ORs) using the Mantel-Haenszel method. Data analysis was performed using Review Manager version 5.4.

**Results:**

Eight studies (4 RCTs and 4 retrospective studies) including 31,047 patients with LACC were included. Mean age was 61.0 years (range: 19–93 years) and mean follow-up was 47.6 months (range: 2–133 months). Of those receiving NAC, 4.6% achieved a pathological complete response and 90.6% achieved R0 resection (versus 85.9%, *P* < 0.001). At 3 years, patients receiving NAC had improved disease-free survival (DFS) (OR: 1.28, 95% confidence interval (CI): 1.02–1.60, *P* = 0.030) and overall survival (OS) (OR: 1.76, 95% CI: 1.10–2.81, *P* = 0.020). When using time-to-effect modelling, a non-significant difference was observed for DFS (HR: 0.79, 95% CI: 0.57–1.09, *P* = 0.150) while a significant difference in favour of NAC was observed for OS (HR: 0.75, 95% CI: 0.58–0.98, *P* = 0.030).

**Conclusion:**

This study highlights the oncological safety of NAC for patients being treated with curative intent for LACC using RCT and propensity-matched studies only. These results refute current management guidelines which do not advocate for NAC to improve surgical and oncological outcomes in patients with LACC.

**Trial registration:**

International Prospective Register of Systematic Review (PROSPERO) registration: CRD4202341723.

**Supplementary Information:**

The online version contains supplementary material available at 10.1007/s00384-023-04482-x.

## Introduction

Traditionally, surgical resection combined with adjuvant chemotherapy (AC) was the cornerstone of managing locally advanced colonic cancer (LACC) [1]. The paradigm of several other gastrointestinal malignancies, including rectal, gastric, and esophageal cancers, has evolved to recognise the benefits of neoadjuvant chemotherapy (NAC) [2–4]. NAC is advantageous for several reasons: tumour downstaging to facilitate complete resection (R0) [5], reducing the theoretical risk of micrometastatic dissemination of cancer cells within human circulation [6], providing in vivo data with respect to sensitivity of the tumour to systemic therapies (recognised to carry prognostic significance) [7], and ensuring higher systemic treatment completion rates (with rationale that complications and postoperative morbidity following surgery may delay progression) [8]. Notwithstanding these perceived benefits of NAC, there remains hesitancy among expert consensus guidelines, such as the National Comprehensive Cancer Network (NCCN) [9], the European Society for Medical Oncology (ESMO) [10], and the National Institute for Health and Care Excellence (NICE) [11], to alter recommendation in support of NAC as the standard of care for LACC. This is likely due to concern regarding overtreatment. Toxicity associated with NAC has been shown to compromise fitness of certain patients due to proceed to surgical resection [12]. Risk of resistant tumours advancing while on NAC is of considerable concern to the multidisciplinary team (MDT) [13]. Thus, contemporary clinical guidelines do not currently advocate for NAC in the setting of LACC.

The FOXTROT trial (NCT00647530) is the largest prospective multicentre randomised clinical trial (RCT) which formally evaluates the value of NAC in radiologically confirmed T3 (≥ 5-mm invasion beyond the muscularis propria) or T4 (tumour penetrates to the surface of the visceral peritoneum and further) LACC [14, 15]. Participants were randomised to either neoadjuvant FOLFOX (5-fluorouracil, leucovorin and oxaliplatin with the addition of panitumumab made based on *Ras* status) followed by surgical resection and subsequent AC or surgical resection followed by 24 weeks of systemic therapy in the adjuvant setting. Preliminary data from FOXTROT illustrated the oncological safety of NAC for patients with LACC, through enhanced survival outcomes, increased R0 resection rates, with lower treatment toxicities, and perioperative morbidity observed for the majority [15].

While FOXTROT provides a degree of optimism surrounding NAC for LACC [14, 15], there remains a paucity of high-quality studies providing long-term data supporting this therapeutic strategy, with several previous analyses failing to randomise or match patients to reduce the natural risk of competing confounding, selection, and ascertainment biases influencing results observed [5, 16]. Accordingly, the aim of the current study was to perform a systematic review and meta-analysis of RCTs and propensity-matched studies to evaluate the oncological safety of NAC in patients being treated with curative intent for LACC.

## Methods

This systematic review was conducted in accordance with the preferred reporting items for systematic reviews and meta-analyses (PRISMA) guidelines, as previously outlined by Moher et al. [17]. As this study used data from previously published studies, ethical approval was not sought from the local institutional review board. All authors contributed to formulating a predetermined review protocol which was then prospectively registered and published on the International Prospective Register of Systematic Reviews (PROSPERO: CRD42023417231).

### Population, intervention, comparison, and outcome (PICO) tool

Applying the PICO framework [18], as previously described by Richardson et al., the research question the authors sought to address through this analysis was as follows:

- Population: any patient diagnosed with radiologically confirmed T3 (defined as disease extending ≥ 5-mm invasion beyond the muscularis propria or similar) or T4 (defined as tumour penetrating to the surface of the visceral peritoneum and other adjacent organs or similar) colon cancers. Patients either had to be randomised (in the clinical trial setting) or indicated to undergo NAC and subsequently matched with similar patients who had undergone upfront surgery followed by AC (in the retrospective cohort studies where propensity matching has occurred).
- Intervention: any patient randomised or indicated to undergo NAC for primary treatment of their LACC.
- Comparison: any patient randomised or indicated to undergo surgery and AC for primary treatment of their LACC.
- Outcomes: the primary outcome measures and study endpoints included the following:
  - Disease-free (DFS) and overall survival (OS) outcomes for patients who underwent NAC and OS, expressed as dichotomous outcomes at 2-year, 3-year, 5-year follow-up and for overall outcomes, or as time-to-effect models as hazard ratios (HRs), with associated 95% confidence intervals (95% CIs).
  - Complete resection (or R0) rates between patients who underwent NAC and AC, expressed as dichotomous outcomes.

### Study definitions

- Overall survival: freedom from mortality due to any cause following treatment for primary LACC [19]
- Disease-free survival: freedom from invasive disease recurrence or mortality due to any cause following treatment of primary LACC [19]

### Search strategy

A predetermined electronic search was performed by two independent reviewers of the PubMed, Scopus, and Cochrane Library databases on the 29th of December 2022 to assess for relevant RCTs and matched studies which would be suitable for inclusion. The search was performed of all fields under the following headings: (neoadjuvant therapies) AND (colon cancer), under medical subheadings (or MeSH Terms), which were linked by ‘AND’ which operated as a Boolean operator. Included studies were limited to those published in the English language, and the authors elected not to restrict included studies based on year of publication. For retrieved studies, their titles were initially screened, before the study abstracts and full texts were evaluated to identify studies which were deemed appropriate for inclusion.

### Eligibility criteria

Studies were considered eligible if they met the following inclusion criteria: (1) studies to be of prospective randomised or retrospective propensity-matched design to be eligible for inclusion in this study, (2) studies had to compare outcomes in adult patients aged 18 years who were randomised (or indicated) to receive NAC and subsequent surgical resection or upfront surgical resection followed by AC following diagnosis with radiologically confirmed T3/T4 colon cancers, and (3) studies had to report oncological and survival outcomes for those in the NAC and AC groups, respectively (as outlined previously).

Studies were excluded if they satisfied any one of the following exclusion criteria: (1) studies reporting outcomes for diseases other than LACC, (2) studies evaluating outcomes in the setting of diseases other than T3 or T4 LACC, (3) studies not reporting clinical outcomes in relation to NAC versus AC, (4) studies including participants aged 17 years and younger, (5) studies where participants were not randomised or matched, (6) case reports or series with less than 5 patients, or (7) any previous review article.

### Data extraction and quality assessment

Literature search was performed by two independent reviewers (M.G.D. and A.H.A.) using the predesigned search strategy, as outlined previously. Duplicate studies were manually removed. Each reviewer systematically reviewed titles, abstracts, and/or full texts before identifying studies which met inclusion criteria. Retrieved manuscripts then had data pertaining to the study information, study design, patient information, treatment details, survival, and oncological outcomes extracted. Risk of bias assessments of included studies was performed using the risk of bias (ROB) tool for RCTs and risk of bias in non-randomised studies - of interventions (ROBINS-I) for non-randomised studies as appropriate [20, 21], as recommended in the 6th edition of the Cochrane Handbook of Systematic Review of Interventions (version 6.3, 2022) [22]. GRADE (Grading of Recommendations, Assessment, Development, and Evaluations) was also performed [23].

### Statistical analysis

Descriptive statistics were primarily used to determine associations between treatment with NAC and AC with both DFS and OS outcomes using Fisher’s exact test (†), as appropriate [24]. Thereafter, treatment strategies and respective survival outcomes were expressed as dichotomous or binary outcomes, before estimation of survival outcomes using the Mantel-Haenszel method. These survival outcomes were expressed as ORs with corresponding 95% CIs, similar to surgical outcomes (i.e.: R0 resection rates) which were also expressed as odds ratios (ORs). The Mantel-Haenszel method is useful in such instances to demonstrate the overall probability of an outcome for two different treatment exposures (i.e.: NAC vs. AC); however, it is limited in that it fails to demonstrate the influence of such exposures over a period of time [25]. Thus, the impact of treatment strategies on respective survival outcomes was analysed using time-to-effect modelling using generic inverse variance method and expressed as hazard ratios (HRs), to demonstrate the influence of these treatments on survival over time [26]. Random effects modelling was applied to all studies on the basis that significant heterogeneity (*I*^2^) had to existed between studies included in analysis, with heterogeneity determined using *I*^2^ statistics. Symmetry funnel plots were used to assess publication bias. All tests of significance were two-tailed with *P* < 0.050 indicating statistical significance. Descriptive statistics were performed using the Statistical Package for Social Sciences (SPSS) version 26 (International Business Machines Corporation, Armonk, New York). Meta-analysis was performed using Review Manager (RevMan), version 5.4 (Nordic Cochrane Centre, Copenhagen, Denmark).

## Results

### Literature search

**Fig. 1:**
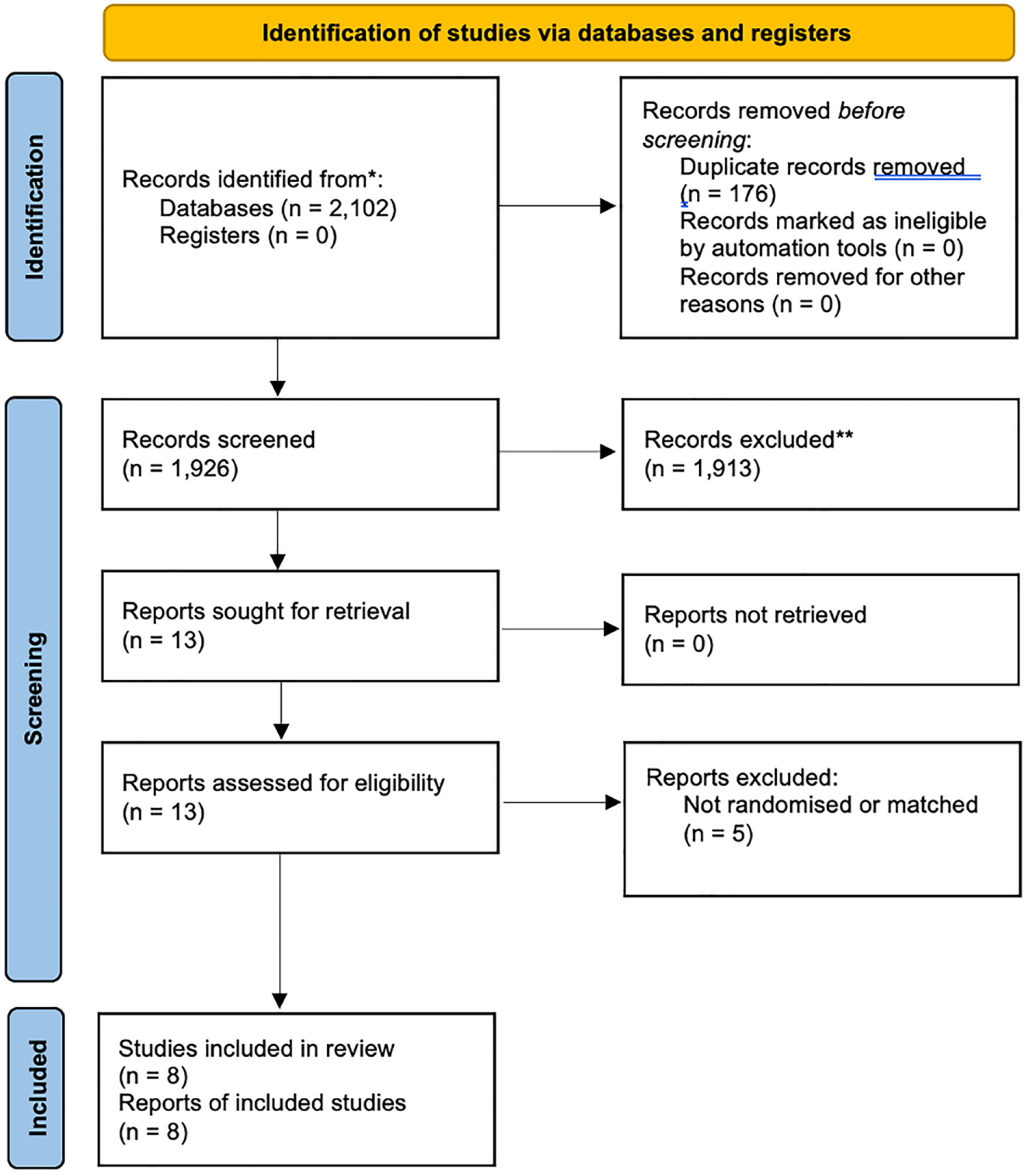
PRISMA flowchart illustrating the systematic search process

Systematic search strategy identified 2102 studies, of which 176 duplicates were manually removed. The remaining 1926 studies had titles screened for relevance, before 30 abstracts and 13 full texts were assessed for eligibility. In total, 8 studies fulfilled inclusion criteria [14, 27–33] (Fig. 1).

### Study characteristics

Of the 8 included studies, 4 were prospective RCTs [14, 27, 29, 33] and 4 were retrospective studies where patients underwent propensity matching [28, 30–32] (both 50.0%). Four of the studies were from European research facilities (50.0%) [14, 28, 29, 32], 2 from China (25.0%) [7, 31], and 1 from both Japan [33] and the USA [30] (both 12.5%), respectively. Publication dates of included studies ranged over a 20-year period (2003–2023). Details and risk of bias assessments from the 8 included studies are outlined in detail in Table 1.

**Table 1:** Details from the 8 studies included in this systematic review

Author	Year	Country	Study design	Regimens	Follow-up (range)	ROB	Grade
Hu	2022	China	RCT	FOLFOX or CAPOX	32.5 months	Low^a^	High
Morton	2023	UK	RCT	OxMdG	37 months	Low^a^	High
de Gooyer	2020	Netherlands	RC - PSM	Various	44 months (4–133)	Moderate^b^	Moderate
Karoui	2020	France	RCT	FOLFOX	54 months	Low^a^	Very low
Dehal	2017	USA	RC - PSM	Various	44 months	Moderate^b^	Low
Zeng	2022	China	RC - PSM	Xelox	62 months (2–83)	Serious^b^	Low
Laursen	2022	Denmark	RC - PSM	Various	-	Moderate^b^	Low
CCCSJG	2003	Japan	RCT	5-FU and MMC	60 months	Low^a^	Moderate

*ROB* risk of bias, *RCT* randomised clinical trial, *RC* retrospective cohort, *PSM* propensity score matched, *UK* United Kingdom, *USA* United States of America, *CCCSJG* Colorectal Cancer Chemotherapy Study Group of Japan, *FOLFOX* leucovorin (folinic acid), 5-fluorouracil, oxaliplatin, *CAPOX/XELOX* capecitabine and oxaliplatin, *OxMdG* oxaliplatin de Gramont, *5-FU* 5-fluorouracil, *MMC* mitomycin C

^a^Use of risk of bias (ROB) tool for randomised clinical trials

^b^Use of risk of bias in non-randomised studies - of interventions (ROBINS-I) tool for non-randomised clinical studies

### Patient characteristics

In total, 31,047 patients who were treated with primary curative intent for T3/T4 LACC were included. The mean age was 61.0 years (range: 19–93 years). Overall, 52.0% were male (16,137/31,047). Mean follow-up was 47.6 months (range: 2–133 months). Patient details from the 8 included studies are outlined in Table 2.

**Table 2:** Patient details from the 8 studies included in this systematic review

Author	Year	Number	*N* NAC	*N* AC	Mean age (range)	Female/male	Stage
Hu	2022	744	371	373	(19–75)	306; 438	T3 (with ≥ 5-mm invasion beyond the muscularis propria or T4)
Morton	2023	1052	698	354	-	-	-
de Gooyer	2020	447	149	298	64 (25–88)	108; 339	T4
Karoui	2020	104	52	52	64 (30–79)	39; 63	High risk T3 or T4
Dehal	2017	27,575	921	26,654	60	13,772; 14,253	T3 or T4
Zeng	2022	126	42	84	67		T3 with ≥ 5-mm invasion beyond the muscularis propria or T4
Laursen	2022	290	145	145	72 (23–93)	-	-
CCCSJG	2003	709	351	358	57	309	Dukes A-D

*CCCSJG* Colorectal Cancer Chemotherapy Study Group of Japan, *N* number, *NAC* neoadjuvant chemotherapy, *AC* adjuvant chemotherapy, *T* tumour stage

### Neoadjuvant chemotherapy characteristics

**Fig. 2:**
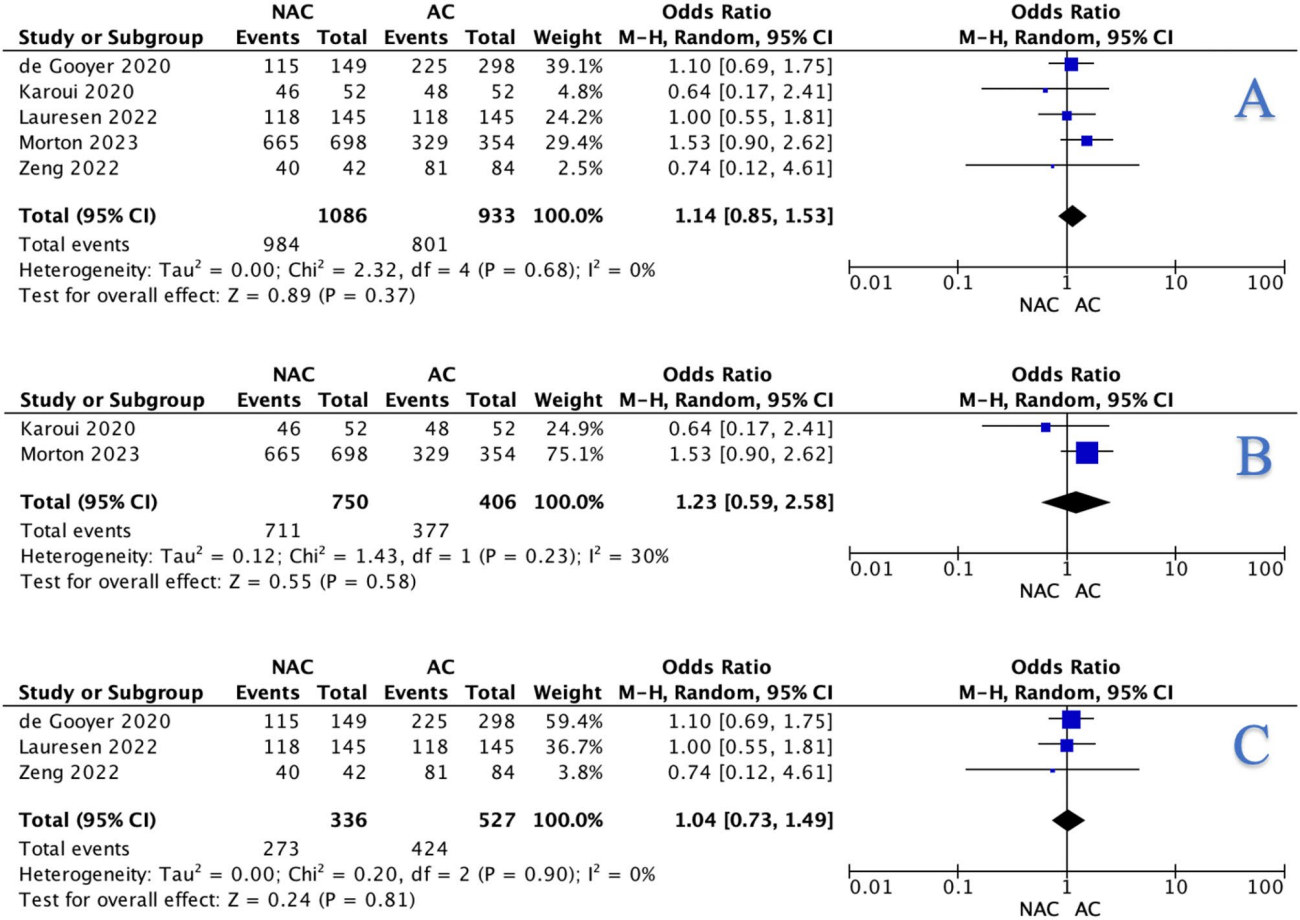
Forest plots illustrating the non-significant difference observed with respect to neoadjuvant chemotherapy and adjuvant chemotherapy for complete resection (R0) rates using the **A** overall data, **B** randomised clinical trial data, and **C** matched data when using the Mantel-Haenszel method

Of the 31,047 patients included in this study, 8.8% were designated to undergo NAC (2729/31,047). In total, 4.6% of those undergoing NAC achieved a pathological complete response (pCR) (67/1457). Overall, 90.6% undergoing NAC (984/1086) and 85.9% in receipt of AC (801/933) achieved an R0 resection (*P* < 0.001, †). At meta-analysis, there was a non-significant difference in R0 resection rates when using overall data (OR: 1.14, 95% CI: 0.85–1.53, *P* = 0.370) (Fig. 2A), RCT data (OR: 1.23, 95% CI: 0.59–2.58, *P* = 0.580) (Fig. 2B), and matched data (OR: 1.04, 95% CI: 0.73–1.49, *P* = 0.810) (Fig. 2C). Details in relation to chemotherapy regimens are outlined in Table 1.

### Disease-free survival

**Fig. 3:**
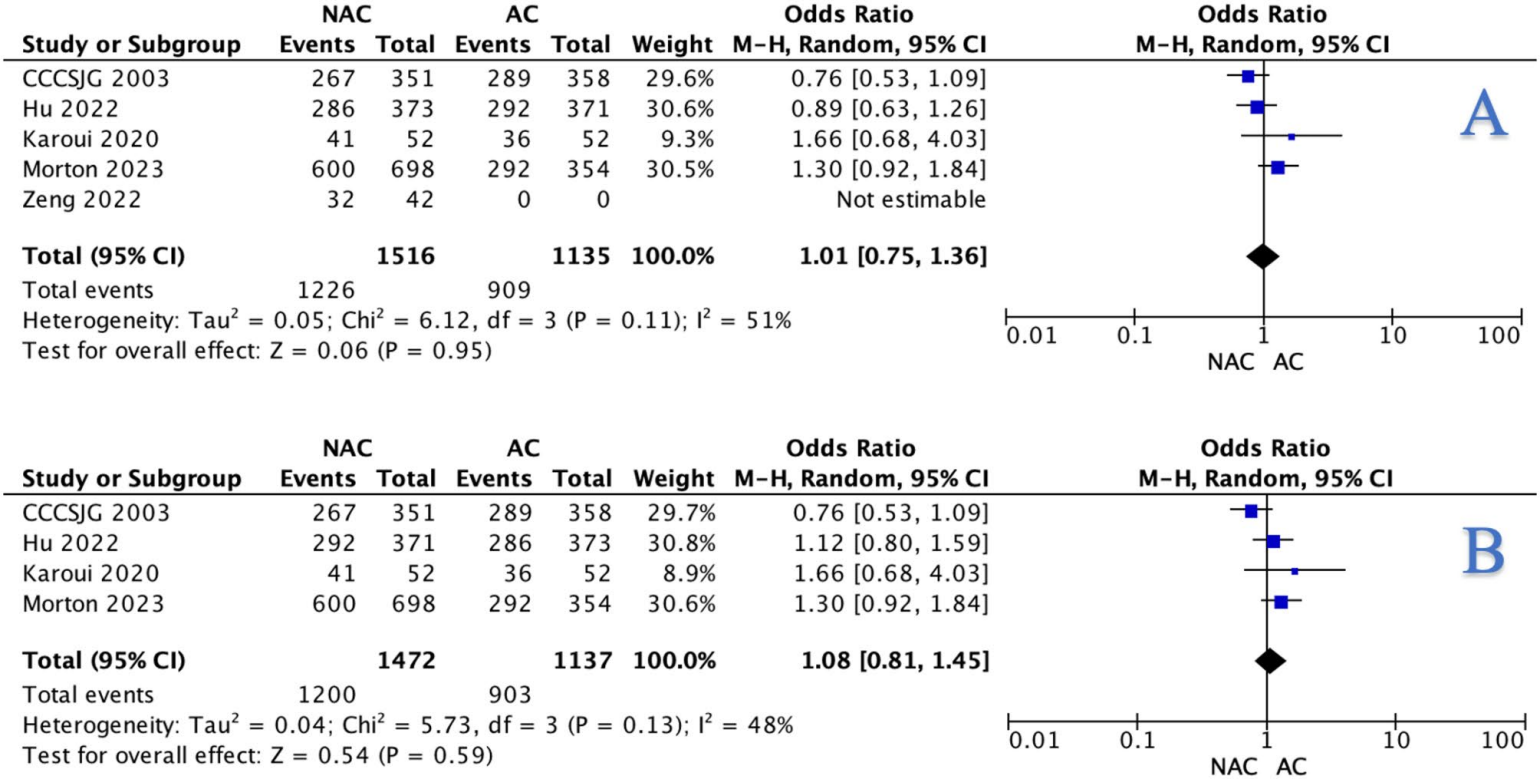
Forest plots illustrating the non-significant difference observed with respect to neoadjuvant chemotherapy and adjuvant chemotherapy for disease-free survival using the **A** overall data and **B** randomised clinical trial data when using the Mantel-Haenszel method

Overall, 81.4% of those undergoing NAC were free of recurrence or death at follow-up (1232/1514) compared to 78.4% of those undergoing AC (957/1221, *P* = 0.005, †) (Table 3). At meta-analysis, this non-significant difference was evident for DFS from overall (OR: 1.01, 95% CI: 0.75–1.36, *P* = 0.950) (Fig. 3A) and RCT data (OR: 1.08, 95% CI: 0.81–1.45, *P* = 0.590) (Fig. 3B). Zeng et al. was the sole study providing matched data for overall DFS and therefore was not analysed at meta-analysis [31].

**Table 3:** Pooled disease-free survival and overall survival data from the 8 studies included in this systematic review

**Parameter**	**NAC**	**AC**	***P***-**value**
R0 resection	984	801	< 0.001^a^†
R1 or R2 resection	202	132	
Overall events	282	264	0.005^a^†
Overall EFS	1232	957	
2-year events	98	62	0.146†
2-year EFS	600	292	
3-year events	211	285	0.001^a^†
3-year EFS	910	594	
5-year events	94	91	0.622†
5-year EFS	299	343	
Overall deaths	444	5725	< 0.001^a^†
Overall OS	2268	22,126	
3-year deaths	299	5517	< 0.001^a^†
3-year OS	1720	21,449	
5-year deaths	85	106	0.458†
5-year OS	308	336	

*NAC* neoadjuvant chemotherapy, *AC* adjuvant chemotherapy, *R0 resection* complete resection, *R1 or R2 resection* incomplete resection, *EFS* event-free survival, *OS* overall survival

^a^Statistical significance

^†^Fisher’s exact test

At 2-year follow-up, 86.0% of those undergoing NAC were free of recurrence or death at follow-up (600/698) compared to 82.5% of those undergoing AC (292/354, *P* = 0.146, †). At 3-year follow-up, 81.2% of those undergoing NAC were free of recurrence or death at follow-up (910/1121) compared to 76.3% of those undergoing AC (594/779, *P* = 0.001, †) (Table 3). At meta-analysis, a significant difference in 3-year DFS was observed in favour of NAC from the overall data (OR: 1.28, 95% CI: 1.02–1.60, *P* = 0.030) (Supplementary Material 1A), which was comprised solely of RCT data.

At 5-year follow-up, 76.1% of those undergoing NAC were free of recurrence or death at follow-up (299/393) compared to 77.6% of those undergoing AC (343/442, *P* = 0.622, †) (Table 3). At meta-analysis, this non-significant difference was evident from the overall data (OR: 1.06, 95% CI: 0.47–2.41, *P* = 0.880) (Supplementary Material 1B).

When using a time-to-effect model at meta-analysis, a non-significant difference was observed from overall (HR: 0.79, 95% CI: 0.57–1.09, *P* = 0.150) (Supplementary Material 1C) and RCT data (HR: 0.82, 95% CI: 0.58–1.15, *P* = 0.250) (Supplementary Material 1D). Once again, Zeng et al. was the only study providing matched data for DFS using time-to-effect modelling and therefore was not analysed at meta-analysis [31].

### Overall survival

**Fig. 4:**
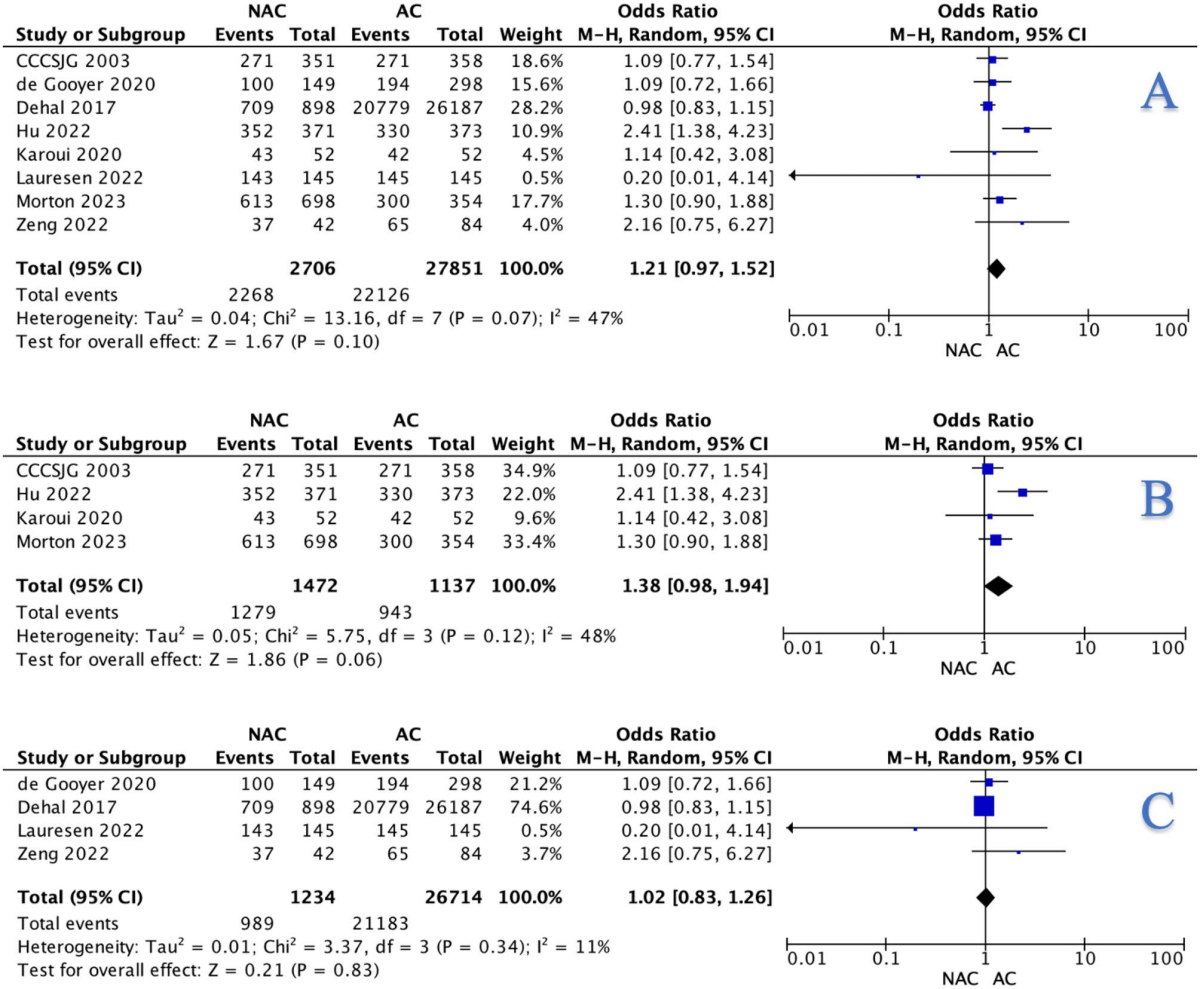
Forest plots illustrating non-significant difference observed with respect to comparison for neoadjuvant chemotherapy and adjuvant chemotherapy for overall survival using **A** overall data, **B** randomised clinical trial, and **C** matched data, respectively, when using the Mantel-Haenszel method

Overall, 83.8% of those undergoing NAC were free of death at follow-up (2268/2706) compared to 79.4% of those undergoing AC (22,216/27,851, *P* < 0.001, †) (Table 3). At meta-analysis, this non-significant difference was evident from the overall (OR: 1.21, 95% CI: 0.97–1.52, *P* = 0.100) (Fig. 4A), RCT (OR: 1.38, 95% CI: 0.98–1.94, *P* = 0.060) (Fig. 4B), and matched data (OR: 1.02, 95% CI: 0.83–1.26, *P* = 0.820) (Fig. 4C), respectively.

At 3-year follow-up, 85.2% of the NAC group were free of death at follow-up (1720/2019) compared to 79.6% of those undergoing AC (21,449/26,966, *P* < 0.001, †) (Table 3). At meta-analysis, a non-significant difference in 3-year OS was observed from the overall (OR: 1.44, 95% CI: 0.94–2.22, *P* = 0.090) (Supplementary Material 2A); however, a significant difference was observed from the RCT data (OR: 1.76, 95% CI: 1.10–2.81, *P* = 0.020) (Supplementary Material 2B).

At 5-year follow-up, 78.3% of those undergoing NAC were free of death at follow-up (308/393) compared to 76.0% of those undergoing AC (336/442, *P* = 0.458, †) (Table 3). At meta-analysis, this non-significant difference was evident from the overall data (OR: 1.27, 95% CI: 0.72–2.22, *P* = 0.410) (Supplementary Material 2C).

**Fig. 5:**
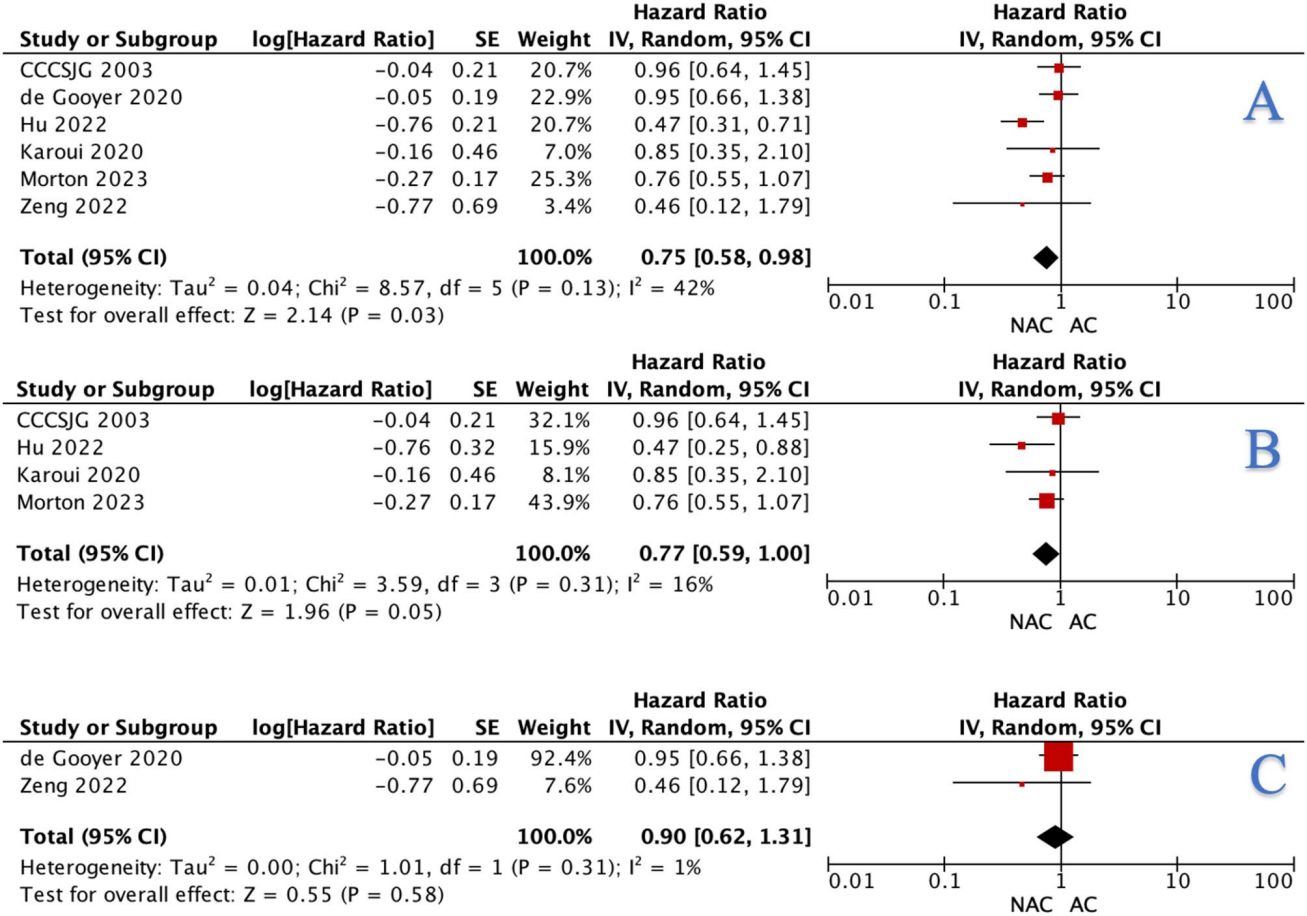
Forest plots illustrating the differences observed with respect to neoadjuvant chemotherapy and adjuvant chemotherapy for overall survival using **A** overall data, **B** randomised clinical trial, and **C** matched data using time-to-effect modelling using the generic inverse variance method

When using a time-to-effect model at meta-analysis, a significant difference was observed from the overall (HR: 0.75, 95% CI: 0.58–0.98, *P* = 0.030) (Fig. 5A) data which was subsequently not apparent from RCT (OR: 0.77, 95% CI: 0.59–1.00, *P* = 0.050) (Fig. 5B) and matched data (HR: 0.90, 95% CI: 0.62–1.31, *P* = 0.580), respectively (Fig. 5C).

## Discussion

This systematic review identified 8 high-quality randomised and propensity-matched studies which provide novel insights into the oncological safety of NAC relative to upfront surgery followed by AC for patients treated with curative intent for LACC. Outcomes from 31,047 patients were included representing some of the most meaningful data regarding NAC use in LACC, since publication of initial results of the seminal FOXTROT trial in 2012 [15]. The most important clinical finding in this analysis is the data supporting NAC as a safe treatment strategy in the setting of LACC, which remained consistent within sensitivity analyses performed using RCT data only. Thus, NAC seems a pragmatic therapeutic strategy which may be utilised in cases of LACC, where deemed feasible by the MDT.

As described, evidence from this study illustrates non-inferiority of NAC relative to AC in LACC, with comparable R0 resection rates and DFS and OS outcomes observed for both treatment arms at meta-analysis. In addition, when comparing raw data for survival between NAC and AC, outcomes tend to significantly favour NAC for both survival metrics (as outlined in detail in Table 3). These are important results, in particular when considered in tandem with 3-year survival outcomes which illustrate a significant improvement in DFS and OS at meta-analysis. Notwithstanding these results losing significance as follow-up progressed beyond the mean follow-up of almost 4 years, more caution should be exercised when interpreting data after this point surrounding true oncological safety of these therapeutic strategies.

Data supporting NAC in LACC is promising, particularly because the management paradigm for LACC has been subject to considerable lag behind other malignancies, where NAC has been adopted as a practical strategy for establishing locoregional control [34–36]. As previously described, the advantages of NAC include increased propensity to achieve tumour downstaging, increased R0 resection rates [5], and theoretical reduction of cancer cells disseminating within human circulation [6] and may be associated with enhanced outcomes [7]. This supports the current analysis where NAC improved surgical and oncological outcomes. Therefore, results of this study support long-term oncological benefit of NAC in LACC, notwithstanding surgical pragmatism of this strategy to improve R0 rates and increasing patient eligibility for local resection [13].

Importantly, the data from the current study and the FOXTROT trial support NAC in T3/T4 LACC [14, 15]. This evidence is not represented in current expert consensus guidelines: Recent NCCN guidelines acknowledge the perceived benefit of NAC in pT4b colon cancer from FOXTROT [9] but fails to endorse NAC in pT4a disease. Moreover, NAC is not recommended by the American Society of Colon and Rectal Surgeons (ASCRS) and European Society for Medical Oncology (ESMO) guidelines for the management of LACC [10, 37], with such therapies currently reserved solely for stage IV disease [38]. Accordingly, this study provides clinical data indicating that NAC is advantageous in LACC, therefore refuting recent recommendations of the aforementioned expert consensuses and guidelines.

Overall, the authors see the data proposed in the current study as being representative of patients with LACC in the ‘real-world’ setting. For example, the mean age of included patients in this study was 61 years, considerably lower than the typical patient with stage IV colon cancer [39], yet directly compared to previous studies where patients received NAC for LACC [5, 40]. Moreover, 52% of patients treated in the current study were male demonstrating consistency with global cancer statistics as reported by Siegel et al. in 2023 [41], where close to a 50:50 split in colon cancer diagnoses was observed among male and female patients with LACC. In addition, a pCR rate of 4.6% was observed in this study, which is similar to previously published study by Hasan et al. from North America which used patient data from the National Cancer Database [42]. Similarly, utility of NAC significantly increased R0 resection rates in this study to approximately 90%, again consistent with recent results of Huang et al. [43]. Accordingly, when considering the promising results of this study in tandem with the work of our colleagues globally, the authors believe this data provides a fair representation of the typical patient who may be subject to NAC as a therapeutic strategy in contemporary LACC management, making these results translatable to clinical practice.

The present systematic review and meta-analysis suffers from a number of limitations. Firstly, various neoadjuvant chemotherapeutic strategies have been evaluated in this study, some of which may be scrutinised in providing limited data within the context of current best practice guidelines. Secondly, while the raw data captured in this study seems to support NAC as a safe treatment in LACC, there is limited data surrounding tumour progression rates, with 10% of patients undergoing incomplete (R1 or R2) resections post NAC. Thirdly, inclusion of retrospective data inevitably renders results subject to unavoidable confounding and selection bias [44]. Fourthly, surgical techniques and concepts surrounding colonic resection have evolved in recent decades (e.g.: complete mesocolic resection) [45], which may have implications on the oncological and surgical outcomes observed for patients with LACC. Finally, it is imperative to highlight that the authors appreciate that under no circumstances, it is sensible to assume that propensity-matched studies are capable of replicating the insights provided by studies of a prospective, randomised design [46]. Thus, their analyses were performed in isolation in the current study as well as being pooled with the RCT data. Nevertheless, as described, the authors believe this data provides ‘real-world’ data with advocacy for use of NAC as a practical therapeutic strategy in LACC.

In conclusion, 8 randomised or propensity-matched studies were identified which, in tandem, highlight the oncological safety of NAC for patients being treated with curative intent for LACC. While this study may face criticism due to inclusion of non-randomised studies, these results certainly refute current management guidelines which do not advocate for NAC as a practical strategy to improve surgical and oncological outcomes in those with LACC.

### Supplementary Information

Below is the link to the electronic supplementary material.

Supplementary file1 (DOCX 553 KB)

## Data Availability

Data will be made available upon responsible request from the corresponding author.
