# Supplementary material for: Evaluating the oncological safety of neoadjuvant chemotherapy in locally advanced colon carcinoma: a systematic review and meta-analysis of randomised clinical trials and propensity-matched studies
Source: Int J Colorectal Dis. 2023 Jul 11;38(1):193. doi: 10.1007/s00384-023-04482-x (PMC10335950; doi:10.1007/s00384-023-04482-x)
Supplement: Supplementary file 1 — Supplementary file1 (DOCX 553 KB) [file 384_2023_4482_MOESM1_ESM.docx]

**Evaluating the Oncological Safety of Neoadjuvant Chemotherapy in Locally Advanced Colon Carcinoma – A Systematic Review and Meta-Analysis of Randomised Clinical Trials and Propensity Matched Studies**

**Supplementary Material**

**Supplementary Material 1.** Forest plots illustrating the comparison for neoadjuvant chemotherapy versus adjuvant chemotherapy for disease-free survival at (A) 3-years and (B) 5-years follow-up respectively, as well as using the (C) overall and (D) randomised clinical trial data through time-to-effect modelling.


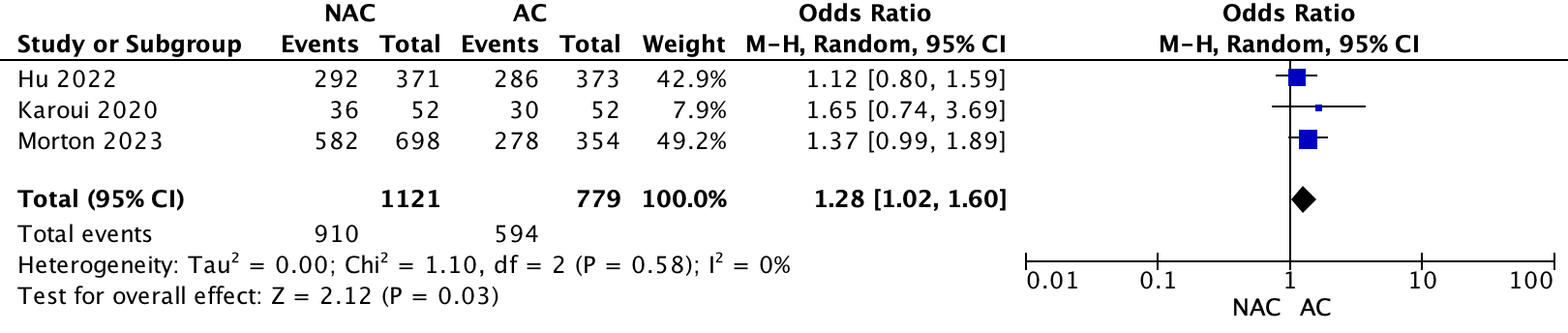


A


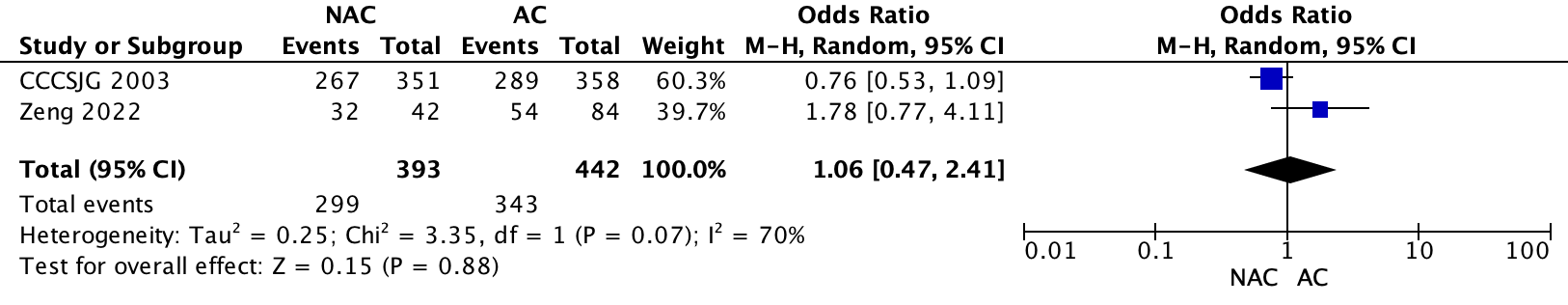


B


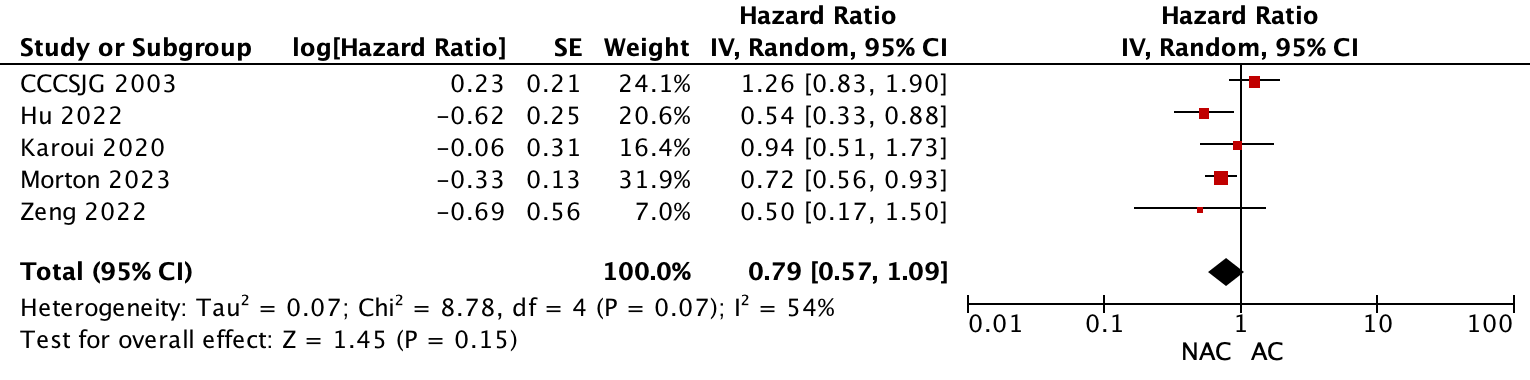


C


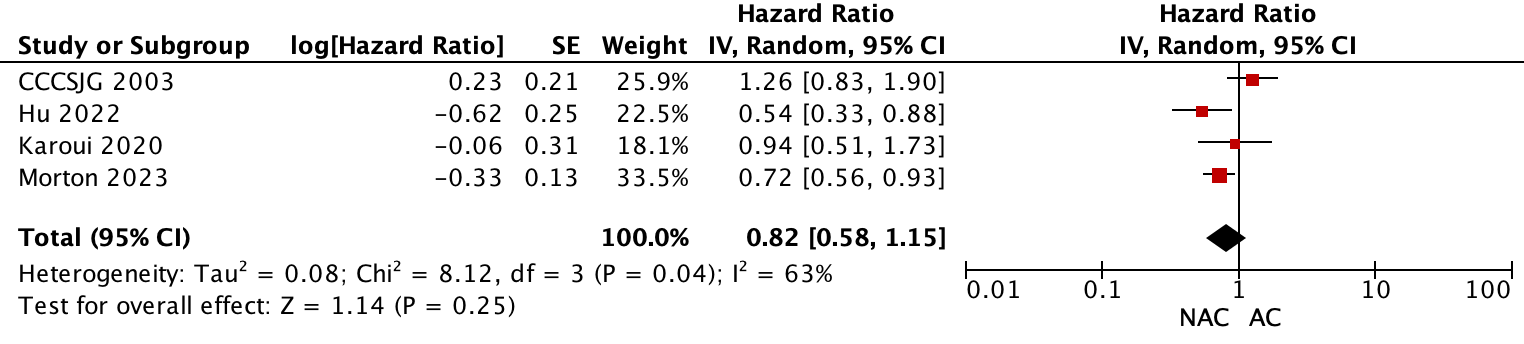


D

**Supplementary Material 2.** Forest plots illustrating the comparison for neoadjuvant chemotherapy versus adjuvant chemotherapy for overall survival at 3-years using (A) overall and (B) randomised clinical trial data respectively, as well as overall survival at 5-years using (C) overall data.


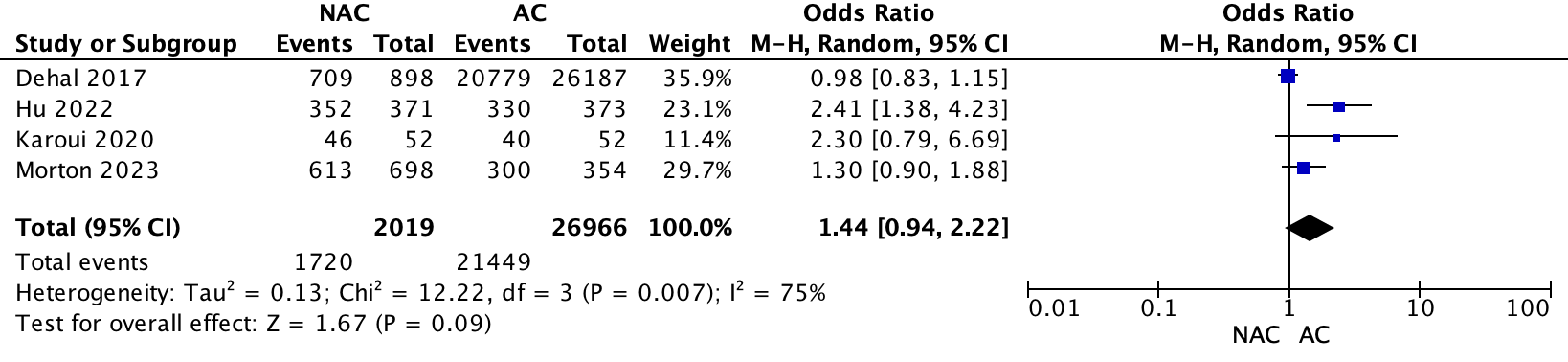


A


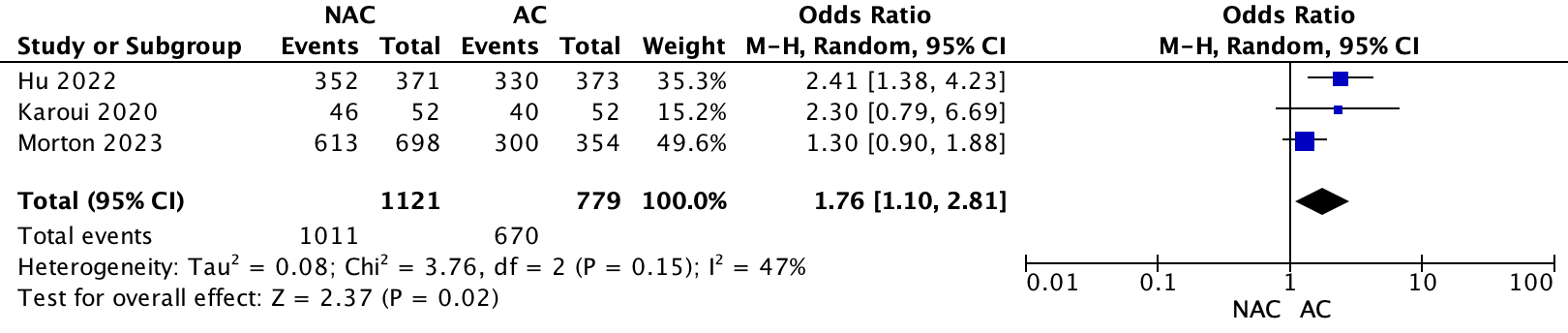


B


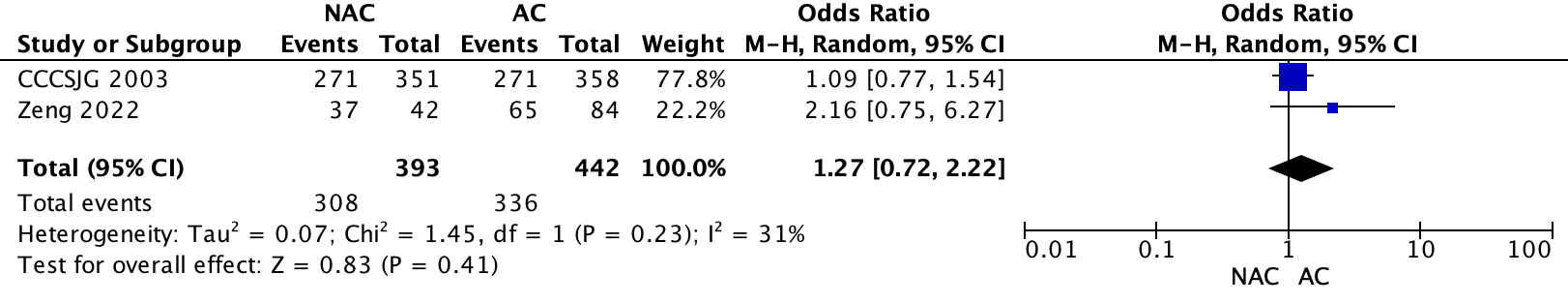


C
